# O Uso Varfarina na Terapia de Anticoagulação Oral: Desafios e Estratégia EmpoderACO para a Promoção do Empoderamento do Paciente no Autocuidado

**DOI:** 10.36660/abc.20230335

**Published:** 2023-06-20

**Authors:** Carina Carvalho Silvestre, Sabrina Cerqueira-Santos

**Affiliations:** 1 GEICF niversidade Federal de Juiz de Fora Governador Valadares MG Brasil Grupo de Estudos Interdisciplinar em Cuidado Farmacêutico (GEICF) – Universidade Federal de Juiz de Fora, Campus Governador Valadares, Governador Valadares, MG – Brasil

**Keywords:** Empoderamento para a Saúde, Comportamentos Relacionados com a Saúde, Educação em Saúde, Anticoagulantes, Varfarina

A varfarina é um anticoagulante oral amplamente utilizado para prevenção primária e secundária de tromboembolismo que pertence à classe dos antagonistas da vitamina K.^1,2^ No Brasil, a varfarina ainda é o principal anticoagulante oral distribuído gratuitamente pelo Sistema Único de Saúde.^3^ Segundo o Instituto de Práticas Seguras no Uso de Medicamentos (ISMP - Brasil), a varfarina é classificada como um medicamento de alta vigilância ou potencialmente perigoso, uma vez que possui baixo índice terapêutico e seu uso requer atenção constante para que o medicamento atue na prevenção de eventos tromboembólicos sem aumentar o risco de hemorragias.^2,4^ Ademais, consta como um dos medicamentos que deve ser alvo de ações prioritárias para a prevenção de danos, de acordo com o Terceiro Desafio Global de Segurança do Paciente, da Organização Mundial de Saúde.^2^

O manejo da terapia anticoagulante com a varfarina é um desafio para muitos pacientes. Requer procedimentos considerados complexos, como o doseamento regular da razão normalizada internacional (RNI), a interpretação dos resultados para ajuste de dose do medicamento pela equipe responsável bem como a compreensão adequada dos fatores que podem influenciar na sua efetividade e segurança, como reações adversas e interações com outros medicamentos e alimentos.^2,4^ Destarte, são necessárias estratégias que auxiliem no uso adequado e seguro do medicamento, além de contribuir na compreensão do seu processo de uso e consequente adesão ao tratamento.

O estudo de Barbosa et al.^5^ teve como objetivo construir e validar o protocolo EmpoderACO voltado para a mudança de comportamento de pacientes em anticoagulação oral com varfarina. Os itens presentes na ferramenta foram organizados com base nos cinco passos presentes no Protocolo Mudança de Comportamento.^6,7^ A construção e validação do protocolo EmpoderACO seguiram etapas bem definidas, propostas pelos estudos de Coluci et al.,^8^ e Pasquali,^9^ Inicialmente, um mapa conceitual foi construído para a identificação dos domínios do autocuidado em anticoagulação oral. O desenvolvimento do instrumento incluiu a participação de um comitê de especialistas interno e externo, composto por diferentes profissionais da área da saúde. Após esta etapa, o protocolo foi submetido para avaliação da validade de conteúdo pelo Comitê de Juízes, seguido da realização do pré-teste em pacientes em uso de varfarina.

A versão final do instrumento resultou em 27 itens, compreendendo diferentes domínios do autocuidado na anticoagulação oral. O protocolo EmpoderACO poderá ser aplicado na prática clínica como suporte no cuidado ao paciente sendo utilizado pelos profissionais de saúde a fim de fortalecer a qualidade das intervenções e estimular o empoderamento do usuário sobre sua farmacoterapia. O envolvimento do paciente no seu processo de cuidado é um dos componentes centrais do cuidado centrado na pessoa, que se relaciona à parceria do profissional com o usuário, baseada na interação e no conhecimento das necessidades e problemas vividos por este. O cuidado centrado na pessoa pretende enfatizar a ideia de que o profissional não deve se ater a investigar apenas as doenças, mas também atender às necessidades do indivíduo de forma ampliada, considerando sua subjetividade.^10^

O cuidado desenvolvido no manejo da anticoagulação oral com a varfarina, especialmente no contexto da atenção primária à saúde, requer um tempo demasiado de acompanhamento no serviço de saúde, bem como a construção de uma relação de confiança com o profissional, a fim de que o vínculo terapêutico auxilie no alcance dos objetivos com o tratamento e no processo de autoconhecimento do usuário acerca do seu tratamento.^11,12^ Este engajamento requer também a consideração do letramento em saúde dos indivíduos, ou seja, do seu conhecimento, motivação e competências para agir, entender, avaliar e aplicar informações de saúde para decisões relacionadas ao cuidado.^13^

Estudos realizados no Brasil e na Austrália encontraram associação positiva entre baixos níveis de letramento em saúde e piores desfechos relacionados ao manejo da terapia de anticoagulação oral com varfarina.^14,15^ Para além, a revisão sistemática de Kim et al.,^16^ em 2017, a qual avaliou diferentes estratégias de envolvimento do paciente e da família para melhorar a segurança dos medicamentos aponta que as intervenções para o envolvimento do paciente não devem ter função apenas informativa, mas devem incorporar níveis mais altos de engajamento dos pacientes. Estes incluem ações que envolvam o paciente nas tomadas de decisão e comunicação ativa com a equipe de saúde, além do estabelecimento de parceria com os indivíduos no atendimento a fim de que estes se tornem colaboradores no seu processo de cuidado, além da inclusão dos pacientes como membros integrais da equipe de cuidado.

O EmpoderACO contempla aspectos de altos níveis de engajamento e pode ser uma ferramenta auxiliar para o empoderamento e envolvimento do paciente no seu processo de autocuidado. Em adição, futuros estudos podem avaliar a implantação da ferramenta em indivíduos com diferentes características, em cenários distintos e avaliar suas potencialidades e fragilidades para a promoção do uso seguro deste anticoagulante.
