# Supplementary material for: O Uso Varfarina na Terapia de Anticoagulação Oral: Desafios e Estratégia EmpoderACO para a Promoção do Empoderamento do Paciente no Autocuidado
Source: Arq Bras Cardiol. 2023 Jun 20;120(6):e20230335. [Article in Portuguese] doi: 10.36660/abc.20230335 (PMC10344365; doi:10.36660/abc.20230335)
Supplement: Translation

# Translation: Use of Warfarin in Oral Anticoagulation Therapy: Challenges and EmpoderACO Strategy for Promoting Patient Empowerment in Self-Care

**Authors:** Carina Carvalho Silvestre, Sabrina Cerqueira-Santos

**Affiliations:** 1 Brazil Grupo de Estudos Interdisciplinar em Cuidado Farmacêutico (GEICF) – Universidade Federal de Juiz de Fora, Campus Governador Valadares, Governador Valadares, MG – Brazil

**Keywords:** Empowerment for Health, Health Behavior, Health Education, Anticoagulants, Warfarin

Warfarin is an oral anticoagulant widely used for primary and secondary prevention of thromboembolism that belongs to the class of vitamin K antagonists.^1,2^ In Brazil, warfarin is still the main oral anticoagulant distributed free of charge by the Unified Health System.^3^ According to the Institute for Safe Practices in the Use of Medicines (ISMP - Brazil), warfarin is classified as a high-alert or potentially dangerous drug since it has a low therapeutic index and its use requires constant attention for the drug to act in the prevention of thromboembolic events without increasing the risk of hemorrhages.^2,4^ Furthermore, it appears as one of the drugs that should be the target of priority actions for the prevention of damage, according to the Third Global Patient Safety Challenge of the World Health Organization Health.^2^

Management of anticoagulant therapy with warfarin is a challenge for many patients. It requires procedures that are considered complex, such as the regular measurement of the international normalized ratio (INR), the interpretation of the results to adjust the drug dose by the responsible team, as well as the adequate understanding of the factors that may influence its effectiveness and safety, such as adverse reactions and interactions with other drugs and foods.^2,4^ Thus, strategies are needed to help in the proper and safe use of the medication and contribute to understanding its use process and consequent adherence to treatment.

The study by Barbosa et al.^5^ aimed to build and validate the EmpoderACO protocol to change patients’ behavior on oral anticoagulation with warfarin. The items in the tool were organized based on the five steps in the Behavior Change Protocol.^6,7^ The construction and validation of the EmpoderACO protocol followed well-defined steps proposed by the studies by Coluci^8^ and Pasquali.^9^ Initially, a conceptual map was built to identify self-care domains in oral anticoagulation. The development of the instrument included the participation of an internal and external expert committee composed of different health professionals. After this stage, the protocol was submitted for content validity assessment by the Committee of Judges, followed by the performance of the pre-test in patients using warfarin.

The final version of the instrument resulted in 27 items comprising different domains of self-care in oral anticoagulation. The EmpoderACO protocol can be applied in clinical practice as support in patient care, used by health professionals to strengthen the quality of interventions and encourage user empowerment regarding their pharmacotherapy. The patient’s involvement in their care process is one of the central components of person-centered care, which is related to the professional’s partnership with the user based on interaction and knowledge of the needs and problems experienced by the latter. Person-centered care emphasizes the idea that the professional should not only investigate diseases but also meet the needs of the individual more broadly, considering its subjectivity.^10^

The care developed in the management of oral anticoagulation with warfarin, especially in the context of primary health care, requires a long follow-up time in the health service, as well as building a relationship of trust with the professional so that the therapeutic bond helps in achieving the goals with the treatment and in the process of self-knowledge of the user about their treatment.^11,12^ This engagement also requires the consideration of the health literacy of individuals, that is, their knowledge, motivation, and skills to act, understand, evaluate, and apply health information to decisions related to care.^13^

Studies carried out in Brazil and Australia found a positive association between low levels of health literacy and worse outcomes related to managing oral anticoagulation therapy with warfarin.^14,15^In addition, the systematic review by Kim et al.^16^ in 2017, which evaluated different patient and family engagement strategies to improve medication safety, points out that interventions for the patient engagement should not only have an informative function but should incorporate higher levels of patient engagement. These include actions involving the patient in decision-making and active communication with the health team, as well as establishing partnerships with individuals in care to become collaborators in their care process and including patients as integral members of the care team.

EmpoderACO contemplates aspects of high levels of engagement and can be an auxiliary tool for patient empowerment and involvement in their self-care process. In addition, future studies can evaluate the tool’s implementation in individuals with different characteristics in different scenarios and evaluate its strengths and weaknesses to promote the safe use of this anticoagulant.
